# One-step colloidal synthesis of biocompatible water-soluble ZnS quantum dot/chitosan nanoconjugates

**DOI:** 10.1186/1556-276X-8-512

**Published:** 2013-12-05

**Authors:** Fábio P Ramanery, Alexandra AP Mansur, Herman S Mansur

**Affiliations:** 1 Center of Nanoscience, Nanotechnology and Innovation - CeNano2I, Department of Metallurgical and Materials Engineering, Escola de Engenharia, Federal University of Minas Gerais, Bloco 2, Sala 2233, Av. Antônio Carlos, 6627, Belo Horizonte, Minas Gerais 31270-901, Brazil

**Keywords:** Nanoparticle, Quantum dot, Colloid, Biopolymer, Chitosan, Bioconjugates, Nanomaterials, 81.07.Ta; 78.67.Hc; 78.67.Sc; 82.35.Pq

## Abstract

Quantum dots (QDs) are luminescent semiconductor nanocrystals with great prospective for use in biomedical and environmental applications. Nonetheless, eliminating the potential cytotoxicity of the QDs made with heavy metals is still a challenge facing the research community. Thus, the aim of this work was to develop a novel facile route for synthesising biocompatible QDs employing carbohydrate ligands in aqueous colloidal chemistry with optical properties tuned by pH. The synthesis of ZnS QDs capped by chitosan was performed using a single-step aqueous colloidal process at room temperature. The nanobioconjugates were extensively characterised by several techniques, and the results demonstrated that the average size of ZnS nanocrystals and their fluorescent properties were influenced by the pH during the synthesis. Hence, novel 'cadmium-free’ biofunctionalised systems based on ZnS QDs capped by chitosan were successfully developed exhibiting luminescent activity that may be used in a large number of possible applications, such as probes in biology, medicine and pharmacy.

## Background

Since the classic talk from Richard Feynman, titled 'There's plenty of room at the bottom’ , presented on 29 December 1959 at the annual meeting of the American Physical Society (at the California Institute of Technology, USA), introduced the concept of nanotechnology, this technology has evolved at an outstanding pace in practically all areas of sciences [1,2]. To be considered as nanotechnology, nanosized and nanostructured systems should present one or more components with at least one dimension ranging from 1 to 100 nm. In recent years, innovation in nanotechnology and nanoscience for medicine (or nanomedicine) has been a major driving force in the creation of new nanocomposites and nanobioconjugates. Essentially, these materials may bring together the intrinsic functionalities of inorganic nanoparticles and the biointerfaces offered by biomolecules and polymers of natural origin, such as carbohydrates and derivatives, glycoconjugates, proteins, DNA, enzymes and oligopeptides [3-5].

In view of the large number of available alternatives to produce hybrids and conjugates for bioapplications, carbohydrates have been often chosen, due to their biocompatibility, physicochemical and mechanical properties, and relative chemical solubility and stability in aqueous physiological environment [5-8]. Among these carbohydrates, chitosan (poly-β(1 → 4)-2-amino-2-deoxy-d-glucose) is one of the most abundant polysaccharides (semi-processed) from natural sources, second only to cellulose [5-8]. Chitosan is a polycationic polymer that has been broadly used in pharmaceuticals, drug carrier and delivery systems, wound dressing biomaterial, antimicrobial films, biomaterials, food packaging and many applications [5-10]. Chitosan is mainly produced from the alkaline deacetylation of chitin (usually extracted from the shells of marine crustaceans, such as crabs and shrimps), forming a copolymer composed of *N*-acetyl-d-glucosamine and d-glucosamine units available in different grades, depending upon the content of the acetylated moieties [5-8]. The degree of deacetylation (DD) and the molar mass (MM) of chitosan influence its properties, such as solubility in water, mechanical behaviour, chemical stability and biodegradability. Similarly, there are several alternatives of one-dimensional and zero-dimensional nanostructured inorganic materials, such as nanotubes, nanowires, nanorods and quantum dots, that are suitable for conjugation with carbohydrates to produce hybrid nanomaterials for bioapplications [11-13]. Quantum dots (QDs) are ultra-small semiconductor nanocrystals that consist of numbers of atoms in the range of a few thousands. Owing to their reduced dimension, QDs exhibit discrete electronic energy levels that give rise to unique electronic, optical and magnetic properties [13-16]. They have rapidly emerged as a new class of fluorescent nanomaterials for a boundless number of applications, primarily as probes in biology, medicine and pharmacy. Having many advantages over organic dyes, such as broad excitation and resistance to photobleaching, QDs are one of the most exciting tools for use in nanotechnology, nanomedicine and nanobiotechnology areas [13]. However, to be used in biological conditions, QDs must exhibit compatibility to the water-based physiological medium in which the large number of natural macromolecules exist. Therefore, surface chemical engineering of QDs is required to render them water soluble and biocompatible. Surprisingly, reports on the surface bio-functionalisation of QDs with chitosan and its derivatives are scarcely found in the literature [5,17-20], and only recently has the direct synthesis of CdS QDs using chitosan and chemically modified chitosans in aqueous colloidal dispersion been published by our group [17-19]. Despite the noticeable advances in the synthesis of nanohybrids based on the conjugation of QDs and biomolecules, to date, most published studies and commercial QDs are synthesised through the traditional organometallic method and contain toxic elements, such as cadmium, lead and mercury, using organic solvents and ligands (trioctyl phosphine/trioctyl phosphine oxide, TOP/TOPO) at high temperatures. Presently, the most commonly used QDs contain divalent cadmium, widely known as a toxin, due to the accumulation of Cd^2+^ in tissues and organs [13,21,22]. Although Cd^2+^ is incorporated into a nanocrystalline core (as components of low-solubility sulphides or selenides) covered by another semiconductor 'shell’ like ZnS and surrounded by biologically compatible ligands, such as polymers, amino acids, proteins and carbohydrates [23-27], it is still unclear if these toxic ions will impact the use of QDs as clinical luminescent probes for biomedical applications. Consequently, great concern has been raised over the toxicity of QDs made by heavy-metal cores in living cells, animals and humans, and in the environment as the long-term impact is not entirely understood [5,22]. In that sense, 'cadmium-free’ nanomaterials are very promising alternatives, such as zinc compounds [5,28], due to their natural environmental abundance. Zinc divalent cations (Zn^2+^) are commonly found in nature, in forms varying from mineral inorganic sources to several living organisms as crucial metabolic species.

Thus, this research focused on demonstrating the synthesis of ZnS quantum dots directly capped by chitosan using a facile, reproducible and economical single-step aqueous processing method at room temperature. Moreover, the nanohybrid systems were extensively characterised, and the strong influence of pH on the formation of the semiconductor nanocrystals and their fluorescent response was verified. The novel colloidal biofunctionalised water-soluble nanoconjugates made of ZnS-QDs/chitosan are potentially non-toxic and, combined with their luminescent properties, offer great potential for use in various biomedical and environmentally friendly applications.

## Methods

### Materials

All reagents and precursors, zinc chloride (Sigma-Aldrich, St. Louis, MO, USA, ≥98%, ZnCl_2_), sodium sulphide (Synth, São Paulo, Brazil, >98%, Na_2_S · 9H_2_O), sodium hydroxide (Merck, Whitehouse Station, NJ, USA, ≥99%, NaOH), acetic acid (Synth, São Paulo, Brazil, ≥99.7%, CH_3_COOH) and hydrochloric acid (Sigma-Aldrich, St. Louis, MO, USA, 36.5% to 38.0%, HCl), were used as received. Chitosan powder (Aldrich, St. Louis, MO, USA, MM = 310,000 to >375,000 g/mol, DD ≥ 75.0% and viscosity 800 to 2,000 cP, at 1% in 1% acetic acid) was used as the reference ligand. Deionised water (DI-water; Millipore Simplicity™, Billerica, MA, USA) with a resistivity of 18 MΩ cm was used in the preparation of all solutions. All preparations and synthesis were performed at room temperature (23°C ± 2°C) unless specified.

### Synthesis of ZnS quantum dots

ZnS nanoparticles were synthesised via an aqueous route in a reaction flask at room temperature as follows: 2 mL of chitosan solution (1% *w*/*v* in 2% *v*/*v* aqueous solution of acetic acid) and 45 mL of DI-water were added to the flask reacting vessel. The pH value of this solution was adjusted to 4.0 ± 0.2, 5.0 ± 0.2 or 6.0 ± 0.2 with NaOH (1.0 mol.L^-1^). Under moderate magnetic stirring, 4.0 mL of Zn^2+^ precursor solution (ZnCl_2_, 8 × 10^-3^ mol.L^-1^) and 2.5 mL of S^2-^ precursor solution (Na_2_S · 9H_2_O, 1.0 × 10^-2^ mol.L^-1^) were added to the flask (S/Zn molar ratio was kept at 1:2) and stirred for 60 min. The obtained ZnS QD suspensions, referred to as QD_ZnS_4, QD_ZnS_5 and QD_ZnS_6, as a function of the pH of quantum dot synthesis, were clear and colourless, and sampling aliquots of 3.0 mL were collected at different time intervals (after preparation, 20 min, 1 h and 24 h) for UV-visible (UV–vis) spectroscopy measurements that were performed for colloidal stability evaluation.

### Characterisation of the ZnS quantum dots and chitosan capping agent

UV–vis spectroscopy measurements were conducted using PerkinElmer equipment (Lambda EZ-210, Waltham, MA, USA) in transmission mode with samples in a quartz cuvette over a wavelength range of 600 to 190 nm. All experiments were conducted in triplicate (*n* = 3) unless specifically noted, and data was presented as mean ± standard deviation.

Photoluminescence (PL) characterisation of the ZnS-chitosan (CHI) conjugates was conducted based on spectra acquired at room temperature using the Nanodrop 3300 fluoro-spectrometer (Thermo Scientific, UV LED with *λ*_excitation_ = 365 ± 10 nm). The relative activity was calculated by subtracting the backgrounds of the samples without QDs. All tests were performed using a minimum of four repetitions (*n* ≥ 4). In addition, QD colloidal media were placed inside a 'darkroom chamber’ , where they were illuminated by a UV radiation emission bulb (*λ*_excitation_ = 365 nm, 6 W, Boitton Instruments, Porto Alegre, Brazil). Digital colour images were collected of the fluorescence of the QDs in the visible range of the spectrum.

X-ray diffraction (XRD) patterns were recorded using a PANalytical X'Pert diffractometer (Cu-Kα radiation with *λ* = 1.5406 Å, Almelo, The Netherlands). Measurements were performed in the 2*θ* range of 15° to 75° with steps of 0.06°.

Nanostructural characterisations of the QD bioconjugates, based on the images and selected area electron diffraction (SAED) patterns, were obtained using a Tecnai G2-20-FEI transmission electron microscope (TEM; Hillsboro, OR, USA) at an accelerating voltage of 200 kV. Energy-dispersive X-ray (EDX) spectra were collected using the TEM for element chemical analysis. In all the TEM analyses, the samples were prepared by dropping the colloidal dispersion onto a porous carbon grid. The QD size and size distribution data were obtained based on the TEM images by measuring at least 100 randomly selected nanoparticles using an image processing program (ImageJ, version 1.44, public domain, National Institutes of Health).

ZnS-CHI quantum dots were analysed by diffuse reflectance infrared Fourier transform spectroscopy (DRIFTS) method (Thermo Fischer, Nicolet 6700, Waltham, MA, USA) over the range of 400 to 4,000 cm^-1^ using 64 scans and a 2-cm^-1^ resolution. These samples were prepared by placing a droplet of the chitosan solution or ZnS-chitosan dispersions onto KBr powder and drying at the temperature of 60°C ± 2°C for 24 h.

For potentiometric titration studies, dried chitosan (0.20 g) was dissolved in 20 mL of 0.10 mol.L^-1^ HCl with gentle stirring overnight and diluted with 20 mL of DI-water. Under continuous stirring, 100 μL of 0.10 mol.L^-1^ sodium hydroxide solution was added, then allowed to equilibrate, and the pH recorded using a pH meter with a glass electrode (Quimis, Diadema, Brazil). This sequence was repeated until neutralisation of the HCl, and deprotonation of amine groups occurred. DD was calculated using Equation 1 [29]:

(1) $$\mathrm{DD}=\left\{\left[M_{\mathrm{NaOH}}\times \left(V_{2}-V_{1}\right)\times {\mathrm{MM}}_{\mathrm{CHI}}\right]/m_{\mathrm{CHI}}\right\}\times 100$$

where *M*_NaOH_ is the molar concentration of NaOH solution (mol.L^-1^) used to neutralise a solution of *m*_CHI_ (g) of chitosan in 0.1 mol.L^-1^ HCl. *V*_2_ (L) is the volume of NaOH added until neutralisation of the ammonium ions from chitosan, and *V*_1_ (L) is the volume of NaOH added to cause the neutralisation of HCl in excess. MM_CHI_ is the molecular mass of glucosamine units (161 g.mol^-1^).

The extent of protonation (EP_pH_) of chitosan can be calculated from Equation 2:

(2) $${\mathrm{EP}}_{\mathrm{pH}}=100–\left[\%{\mathrm{NH}}_{2}\times \left(100/\mathrm{DD}\right)\right]$$

where% NH_2_ is the amount of non-protonated amine groups estimated from Equation 1 considering that *V*_2_ is equal to the added volume of base to neutralise the ammonium ions from chitosan at the pH of interest (4.0, 5.0 and 6.0).

Zeta potential analyses were performed using a Brookhaven ZetaPALS instrument with a laser light wavelength of 660 nm (35-mW red diode laser, Holtsville, NY, USA). Standard square acrylic cells with a volume of 4.5 mL were used. The zeta potential measurements were performed at (25.0°C ± 2°C) under the Smoluchowski approximation [30], and 100 runs (five measurements of 20 cycles) were chosen for a good reproducibility.

## Results

### Characterisation of ZnS quantum dots capped by chitosan

#### UV–vis spectroscopy

**Figure 1:**
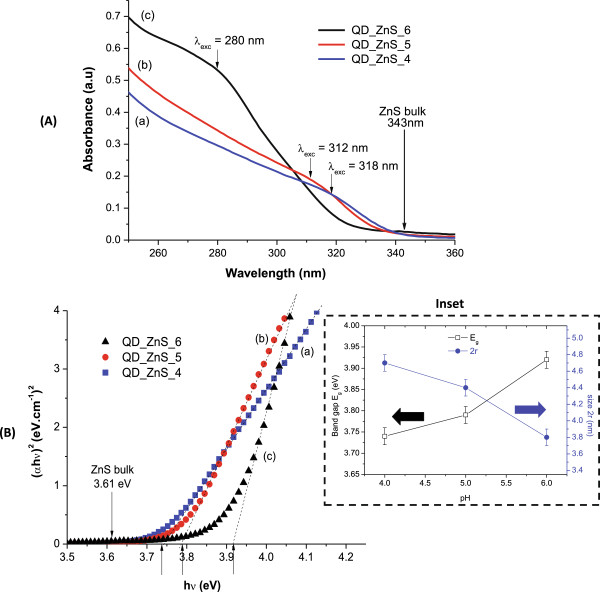
**UV–vis spectroscopy analysis. (A)** Spectra of ZnS-chitosan conjugates synthesised at different pH. **(B)** Optical band gap using the Tauc relation of ZnS-chitosan conjugates synthesised at different pH. (a) pH = 4.0, (b) pH = 5.0, (c) pH = 6.0. Inset: analysis of the effect of pH during the synthesis on the average ZnS quantum dot size (2*r*) and respective band gap energy (*E*_QD_).

The UV–vis absorption spectra of the ZnS nanoparticles produced using chitosan as the stabilising ligand (ZnS-chitosan nanoconjugates) are shown in Figure 1A. The curves exhibit a broad absorption band between 250 and 360 nm associated with the first excitonic transition indicating that ZnS nanocrystals were synthesised within the 'quantum confinement regime’ [31] at different pH to form colloidal suspensions capped by carbohydrate-based ligands (after 24 h). The band gap of quantum dots may be assessed by theoretical, semi-empirical and empirical models. In this study, the optical band gap energy (*E*_QD_) was assessed from absorption coefficient data as a function of wavelength using the 'Tauc relation’ [32]. This procedure allows to estimate the dimensions of nanoparticles in diluted colloidal suspensions *in situ* once the average size of the ZnS nanocrystals can be estimated using the empirical model published in the literature [33,34], which relates the nanoparticle size (*r*) to the *E*_QD_ from a UV–vis spectrum (Equation 3):

(3) $$r\left(E_{\mathrm{QD}}\right)=\left[0.32-2.9\times {\left(E_{\mathrm{QD}}-3.49\right)}^{1/2}\right]/2\times \left(3.50-E_{\mathrm{QD}}\right)$$

The *E*_QD_ values extracted from the curves using the Tauc relation (Figure 1B) were equal to 3.74 ± 0.02, 3.79 ± 0.02 and 3.92 ± 0.02 eV for pH = 4.0, 5.0 and 6.0, respectively. These band gap values are higher than the reference bulk value of 3.54 to 3.68 eV for ZnS with a cubic structure, the difference (*E*_QD_ - *E*_g_) being referred to as 'blue shift’ [35,36]. Additionally, based on *E*_QD_ results, the average sizes (diameter, 2*r*) were calculated (Equation 4) to be 4.7 ± 0.1, 4.4 ± 0.1 and 3.8 ± 0.1 nm for pH = 4.0, 5.0 and 6.0, respectively. Statistical analysis showed that the pH of the synthesis has influenced optical properties and nanoparticle dimensions (Student's *t* test, 95% confidence coefficient; 0.05 significance level), as shown in Figure 1B (inset). The summary of the results extracted from the UV-visible spectra and optical absorbance analysis is presented in Table 1.

**Table 1:** Parameters of ZnS QDs capped by chitosan as a function of pH during the synthesis

**Sample**	**pH**	***λ***_**exc **_**(nm)**	***E***_**QD **_**(eV)**	**Blue shift (eV)**	**Size, 2** ***r *** **(nm)**
				**Bulk**^**a**^ **= 3.61**	
QD_ZnS_4	4.0 ± 0.1	318 ± 2	3.74 ± 0.02	0.13 ± 0.02	4.7 ± 0.1
QD_ZnS_5	5.0 ± 0.1	312 ± 2	3.79 ± 0.02	0.18 ± 0.02	4.4 ± 0.1
QD_ZnS_6	6.0 ± 0.1	280 ± 2	3.92 ± 0.02	0.31 ± 0.02	3.8 ± 0.1

^a^Reference bulk value for ZnS (cubic crystalline structure).

#### Photoluminescence spectroscopy analysis

**Figure 2:**
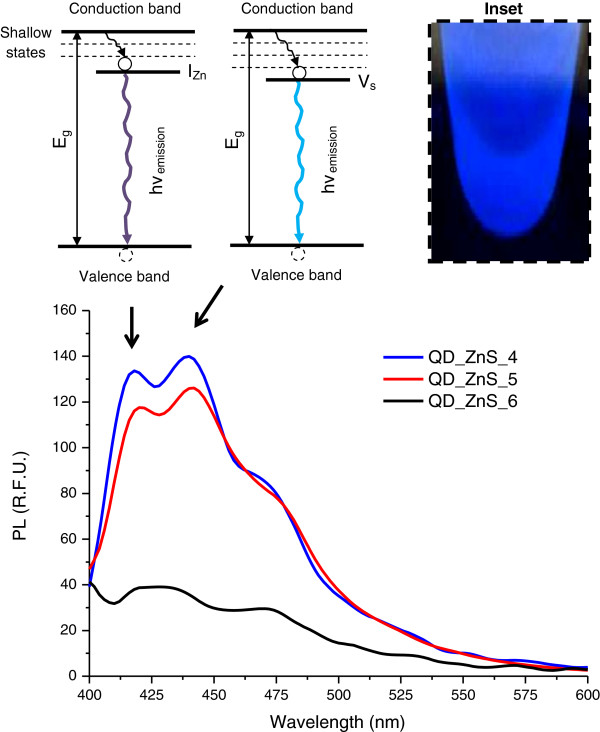
**PL spectra of ZnS-chitosan conjugates at pH = 4.0, pH=5.0, and pH = 6.0.** Inset: blue luminescence under UV excitation.

Based on the absorbance curves and the band gap energies evaluated under excitation, ZnS-chitosan bioconjugates were expected to emit light in the UV range (*E*_g_ ≥ 3.6 eV). However, the occurrence, population and depths of the traps determine the pathway that the electron–hole (e^-^/h^+^) pair generated by the absorption of light will follow, i.e. recombine and produce the emission of light and/or undergo non-radiative decay. ZnS quantum dots typically exhibit emission peaks in the 400 to 550 nm wavelength range that is primarily associated with point defects, such as vacancies (V) and interstitial ions (I) and also surface defects [20,37,38]. The band edge (excitonic) emission from ZnS, being related to more organised and highly crystalline materials, has been sparsely detected [37,38]. Figure 2 shows the photoluminescence spectra collected at room temperature (RT) of the nanoparticle-biopolymer systems under evaluation. From a general perspective, the band edge recombination was not detected, and other bands in the violet-blue range were observed (Figure 2, inset). According to the energy level diagrams reported by Wageh et al. [38] and Becker and Bard [39], the high-energy emission bands (wavelengths below 450 nm) observed in the spectra are associated with the V_s_ (vacancies of sulphur, S^2-^) and I_Zn_ (Zn^2+^ at interstitial sites at the lattice) defects because they may be favoured by the synthesis of the nanoparticles under the condition of an excess of metal atoms, compatible with the procedure used in this work using a stoichiometric molar ratio of Zn^2+^/S^2-^ = 2:1. In addition, because vacancy states lie deeper in the band gap than do the states arising from interstitial atoms in colloidal ZnS [38-40], the emission band of QD_ZnS_4 and QD_ZnS_5 identified at about 418 nm (2.97 eV) is due to transitions involving interstitial states, while the emission around 440 nm (2.82 eV) is assigned to vacancy states. The band at approximately 470 nm (2.63 eV), observed for all systems (independent to the pH of the synthesis), may be assigned to surface defects [38].

#### XRD analysis

**Figure 3:**
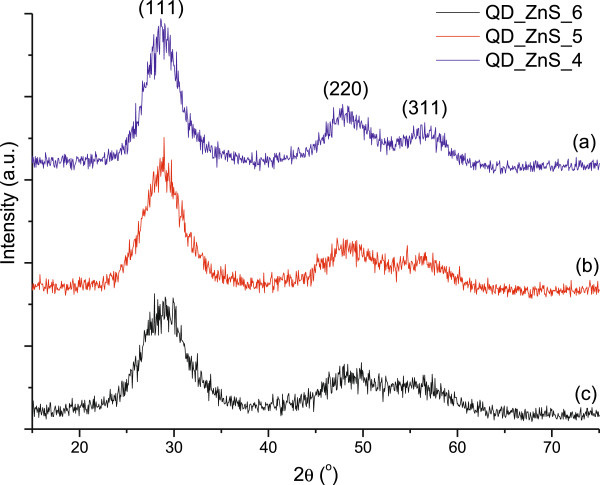
**XRD patterns of ZnS quantum dots synthesised at different pH. (a)** pH = 4.0, **(b)** pH = 5.0, **(c)** pH = 6.0.

The XRD patterns of ZnS QDs prepared at different pH have presented similar peak profiles, with a relative increase of the peak broadening related to the rise of the pH of QD preparation (Figure 3). The three peaks observed in the patterns at 2*θ* ~ 28.7°, 2*θ* ~ 48.0° and 2*θ* ~ 56.3° could be assigned to the planes (111), (220) and (311) of ZnS of the cubic lattice structure (zinc blend also referred to as sphalerite, JCPDS 05–0566). This crystalline form has been reported by several authors for nanoparticles of ZnS, despite hexagonal wurtzite being the stable polymorph of ZnS bulk at ambient temperatures [41-43]. The peak broadening observed in XRD patterns is associated with the formation of small crystals [41,43]. Besides, for the smaller particles, the peak broadening is larger and peaks overlap in a large extent. Based on these features, the obtained XRD profiles are in accordance with the results of nanoparticle dimensions estimated by UV–vis spectra with the smaller crystallite size related to the higher pH of the synthesis.

#### TEM morphological analysis

**Figure 4:**
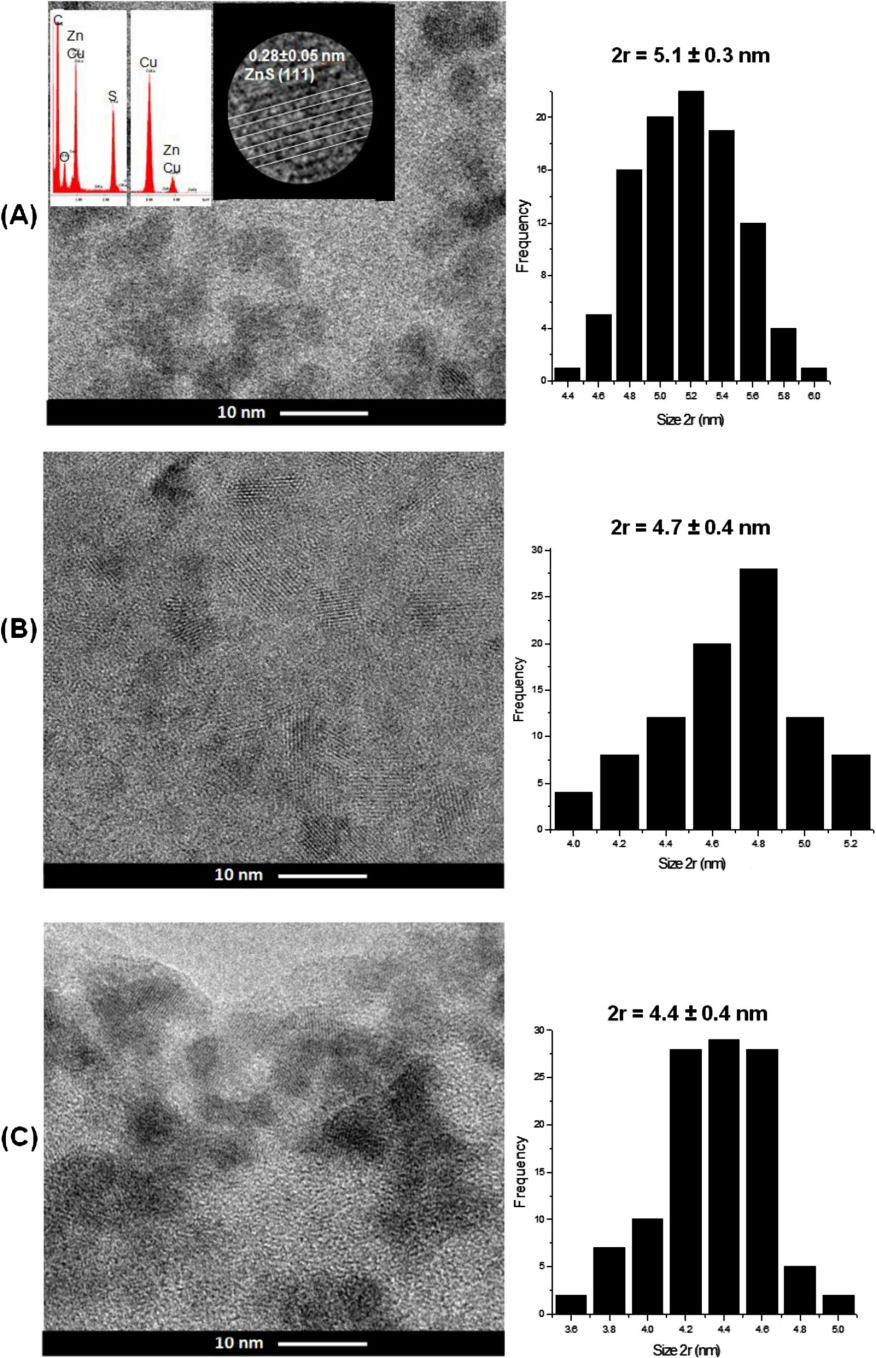
**TEM and EDX analysis. (A)** TEM image and particle size distribution histogram of QD_ZnS_4 bioconjugates. Inset: EDX spectrum and nanocrystal plane spacing. TEM images and particle size distribution histograms of **(B)** QD_Zn_5 and **(C)** QD_ZnS_6.

In this study, the morphological and structural features of the quantum dots were characterised using TEM coupled to an EDX microprobe and using SAED analysis. Figure 4 shows representative samples of ZnS QDs produced with the chitosan at pH 4.0 ± 0.2 (A), pH 5.0 ± 0.2 (B) and pH 6.0 ± 0.2 (C) with spherical shape. EDX spectra show the chemical analysis of the nanocrystals with Zn and S as the major elements (Figure 4A, inset), excluding the copper, oxygen and carbon peaks related to the TEM grid and the polymer stabiliser. The electron diffraction pattern of the QDs with a lattice parameter comparable to the ZnS cubic crystal (JCPDS 05–0566) is shown in Figure 4A (inset). The histogram of the QD_ZnS_4 size distribution (Figure 4A) indicates a monodisperse distribution with an average size of 5.1 ± 0.3 nm. Analogously, QD_ZnS_5 and QD_ZnS_6 samples exhibited reasonably monodisperse nanoparticles, with an average size centred at approximately 4.7 ± 0.4 nm (Figure 4B) and 4.4 ± 0.4 nm (Figure 4C), respectively. Thus, the TEM results demonstrated that ZnS quantum dots were properly stabilised by chitosan, in reasonable agreement with the values obtained from the UV–vis optical absorbance in the previous section for QD_ZnS_4 (2*r* = 4.7 ± 0.1 nm), QD_ZnS_5 (2*r* = 4.4 ± 0.1 nm) and QD_ZnS_6 (2*r* = 3.8 ± 0.1 nm).

#### FTIR spectroscopy analysis

Fourier transform infrared (FTIR) spectroscopy is commonly used to better understand the local nano-microenvironment of the ligands at the QD surface. In some cases, it has proven to be the most important technique for the characterization of the interactions between the ligand and the quantum dot [35,44]. The FTIR spectrum of chitosan copolymer (Additional file 1: Figure S1) presents absorption peaks at 1,645 and 1,560 cm^-1^ which are assigned to the carbonyl stretching of the secondary amides (amide I band) and the N-H bending vibrations of the deacetylated primary amine (-NH_2_) and amide II band, respectively. NH vibrations (stretching) also occur within the 3,400 to 3,200 cm^-1^ region overlapping the OH stretch from the carbohydrate ring. In addition, the absorptions at 1,030 to 1,040 cm^-1^ and 1,080 to 1,100 cm^-1^ indicate the C-O stretching vibration in chitosan, which are associated with the C6-OH primary alcohol and the C3-OH secondary alcohol, respectively [6,19,45]. These amine, amide and hydroxyl groups are the most reactive sites of chitosan and are involved in the chemical modifications of this carbohydrate and in the interactions of chitosan with cations and anions [46,47].

**Figure 5:**
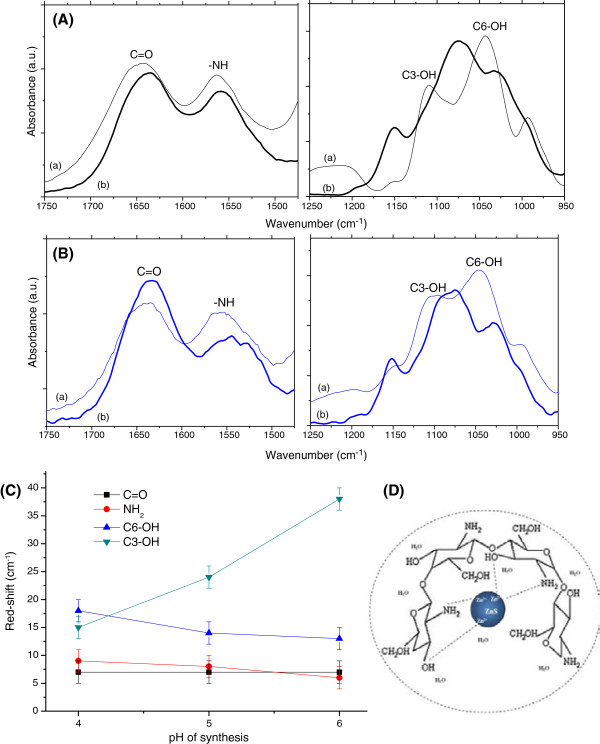
**FTIR spectra results.** (a) Chitosan and (b) ZnS-chitosan bioconjugates at **(A)** pH 6.0 ± 0.2 and **(B)** pH = 4.0 ± 0.2. Vibrational regions: 1,750 to 1,475 cm^-1^ (left) and 1250–950 cm^-1^ (right). **(C)** Relative 'red-shift’ of bands associated with the functional groups of chitosan after the formation of ZnS bioconjugates as a function of pH. **(D)** Schematic representation of some interactions at ZnS-chitosan nanointerfaces (not to scale).

After conjugating the quantum dots with the capping biopolymer (curves (b) in Figure 5 and Additional file 2: Figure S2), there were several bands of chitosan in the FTIR spectra (curves (a) in Figure 5 and Additional file 2: Figure S2) that exhibited changes in their energies (i.e. wavenumber). These changes can be mainly attributed to the interactions occurring between the functional groups of the chitosan ligand (amine/acetamide and hydroxyls) and the ZnS QDs. For example, in the spectra of the bioconjugated QDs (Figure 5), the amide I band (1,650 cm^-1^) shifted to a lower wavenumber by 7 cm^-1^ for the ZnS nanoconjugates synthesised at pH 4.0 and 6.0. The amine band (bending NH, at 1,560 cm^-1^) was 'red-shifted’ (i.e. shifted to a lower energy) by approximately 6 cm^-1^ for QD_ZnS_6 and 9 cm^-1^ for QD_ZnS_4. A significant change was also observed in the region from 1,000 to 1,200 cm^-1^, which was essentially associated with -OH groups (alcohol groups). The band associated with the primary alcohol (C6-OH) vibration was red-shifted by 13 cm^-1^ for QD_ZnS_6 and 18 cm^-1^ for QD_ZnS_4. The peak assigned to C3-OH (secondary alcohol) stretching shifted its position to a lower energy by 38 cm^-1^ for QD_ZnS_6 and 15 cm^-1^ for QD_ZnS_4. Figure 5C summarises the red shift of bands related to functional groups of chitosan after bioconjugation as a function of pH. Additionally, at all the pH concentrations under evaluation, the wide peak of chitosan at 3,385 cm^-1^ (Additional file 3: Figure S3), corresponding to the stretching vibration of -NH_2_ and -OH groups, became significantly narrower after stabilisation of the quantum dots. This peak narrowing indicates the reduction of 'free’ amine groups after quantum dot stabilisation [35].

Based on the FTIR analyses, the primary and secondary alcohols and the amine and acetamide (carboxyl) groups in chitosan were determined to have interacted with the ZnS quantum dots. The differences between the FTIR spectra of chitosan before and after conjugation with ZnS nanocrystals can be assigned to the formation of coordination complexes between chitosan and zinc cations (Zn^2+^) on the surfaces of the QDs, with the participation of the amino and/or hydroxyl functional groups, besides carboxyl groups from acetamide [44,48,49]. Metal ions have been suggested to be chelated with the NH_2_, OH and NH-CO-CH_3_ groups in the chitosan chain as mono- and/or multidentate ligands (Figure 5D), depending on the type and concentration of the metal species, the functional derivative groups and the pH level [47,49,50].

### Characterisation of the chitosan capping agent

From the curve of the potentiometric titration of chitosan (Additional file 4: Figure S4), the DD was calculated to be equal to 75% ± 2% (in accordance with the specification from the manufacturer, ≥75.0%), and EP_pH_ was estimated to be 100%, 92% and 60% at pH levels of 4.0, 5.0 and 6.0, respectively, which are consistent with previous studies reported in the literature [51].

**Figure 6:**
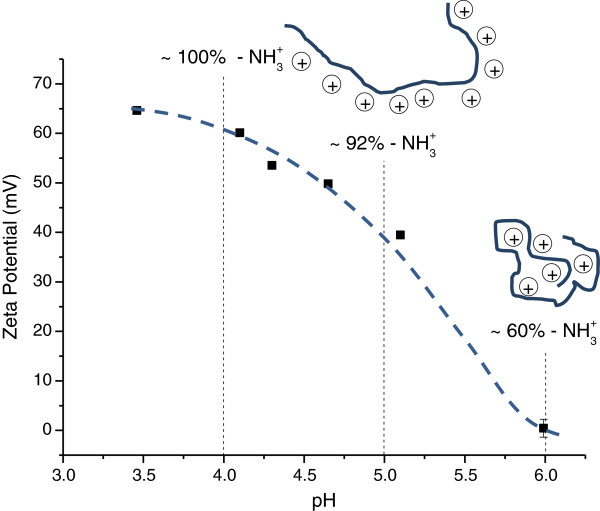
**Zeta potential curve of chitosan solutions at different pH.** Calculated values of the 'extent of protonation’ with the respective schematic representation of chitosan polymer conformation/charges (range from 3.5 to 6.0).

Aiming at a more in-depth investigation, the characterisation of the chitosan by zeta potential measurements was performed, thus providing information on the possible chemical interactions occurring at the chitosan-quantum dot interfaces. Figure 6 shows the zeta potential of the chitosan solutions at different pH levels with EP_pH_ data. These results indicated a decrease of the surface charge with an increasing pH level ranging from +65 mV at pH 3.5 to approximately 0 mV close to pH 6.0. These results follow the same trend as that of the extent of protonation as a function of pH: a higher potential zeta value was measured for a higher content of -NH_3_^+^ groups, as depicted in Figure 6.

## Discussion

The UV–vis absorption spectra were used to monitor the formation of ZnS QDs capped with chitosan and also to calculate some optical properties of these nanocrystals. The results of *E*_QD_ of the ZnS QDS synthesised at different pH were larger than that of the original bulk material (*E*_g_), demonstrating that semiconductor nanoparticles with dimensions below the 'Bohr radius’ were produced. It should be highlighted that these results are notably relevant as far as the direct synthesis of semiconductor nanocrystals in aqueous media is concerned because ZnS QDs were nucleated and stabilised at ultra-small sizes using a water-soluble biocompatible polysaccharide. In fact, to the best of our knowledge, this is the first report where cadmium-free bioconjugates based on ZnS QDs were directly produced and stabilised by chitosan at room temperature using strictly water colloidal chemistry. To obtain these results, the carbohydrate ligand must cap and stabilise the ZnS nuclei at the very early stages of the reaction that formed the water colloidal suspensions. Moreover, the ZnS nuclei should have surpassed the thermodynamic factor for growing the QD nuclei and agglomeration that is driven by the minimisation of the system surface energy. The kinetic aspects of the reaction of Zn^2+^ with S^2-^ for producing ZnS nanocrystals must be considered as very favourable, due to the free energy (Δ*G* < 0), and a 'burst of nuclei’ is observed due to the high reaction rate (i.e. very low 'solubility product constant’ , *K*_sp_ = ~10^-24^) [52].

From the perspective of using chitosan as the stabiliser ligand, additional considerations may be drawn regarding the formation of ZnS nanocrystals. Chitosan is considered to be a pH-sensitive polymer and a weak base in aqueous solutions, with a pKa value of approximately 6.5 [53]. This pKa value leads to the protonation of the amine groups in acid solutions according to Equation 4:

(4) $${{\mathrm{CHI}‒\mathrm{NH}}_{2}}_{\left(\mathrm{aq}\right)}+{H^{+}}_{\left(\mathrm{aq}\right)}\to \mathrm{CHI}‒{\mathrm{NH}}_{3}{{}^{+}}_{\left(\mathrm{aq}\right)}$$

Considering Equation 4 and the results presented in Figure 6, under acidic conditions (pH < pKa), the amine group of chitosan is protonated to various degrees, depending on the pH of the solution: the lower the pH value (referenced to pKa), the higher the extension of the protonation (NH_2_ → NH_3_^+^). However, note that despite the presence of the protonated groups, the surface charge of chitosan at pH 6.0 tends towards zero, which could be due to the conformation of the chitosan chains. At lower pH levels, almost all of the amine groups are protonated, thus repealing each other and thereby favouring the chitosan-water interaction, which overcomes the associative forces between chains. At higher pH levels, the number of -NH_3_^+^ species and the net of the interchain repulsive electrostatic forces are reduced. Hydrogen bonds and hydrophobic interactions between chains will be more favourable, thus promoting the formation of a more compact structure [54,55].

As a consequence, a significant influence of pH on the formation/growth/stabilisation and optical properties of the ZnS QDs in chitosan colloidal solution was observed (as depicted in Figure 1B, inset). Based on the UV–vis spectroscopy results, when the pH was raised from 4 to 6, the average nanocrystal size decreased by approximately 20% (from 4.7 to 3.8 nm). Additionally, a similar trend was observed from XRD and TEM analyses: an increase in the pH of the medium caused a reduction on the average QD size. The decrease in size could be attributed to the sum of several contributions towards the formation of the nanoconjugates made by the ZnS 'core’ and chitosan 'shell’. At a relatively lower pH (pH = 4), most of the amine groups of chitosan are protonated (pH < < pKa of chitosan); thereby, positively charged transition metal has to compete with hydrogen ion for complexation with amine electron pair (metal-ligand interactions), as represented in Equations 5 and 6 [50]:

(5) $$\mathrm{Chitosan}‒{\mathrm{NH}}_{2\left(\mathrm{aq}\right)}+{H^{+}}_{\left(\mathrm{aq}\right)}\to \mathrm{Chitosan}‒{\mathrm{NH}}_{3}{{}^{+}}_{\left(\mathrm{aq}\right)}$$

(6) $$\begin{array}{ll} \mathrm{Chitosan}‒{\mathrm{NH}}_{3}{{}^{+}}_{\left(\mathrm{aq}\right)} & +{{\mathrm{Zn}}^{2+}}_{\left(\mathrm{aq}\right)}\to {{\left[\mathrm{Chitosan}‒{\mathrm{NH}}_{2}/\mathrm{Zn}\right]}^{2+}}_{\left(\mathrm{aq}\right)}\\ & +{H^{+}}_{\left(\mathrm{aq}\right)} \end{array}$$

However, as the pH increases (pH = 6), more amine groups become available in the chitosan chain for dative bonding (electron donor) with zinc divalent cations, thus reducing the electrostatic repulsion (Zn^2+^ ↔ NH_3_^+^) and favouring the stabilisation of the ZnS nanocrystals at smaller dimensions due to the increase of the number of nucleation sites.

It is also interesting to note that the shift of the secondary alcohol vibration in FTIR spectra of conjugates was inversely proportional to the extent of protonation. Both the amine/protonated amine and the C3-OH group are at the same side of the chitosan chain. The presence of a higher number of -NH_3_^+^ charged groups may affect the interaction of -OH groups with metal cations (Zn^2+^) during the nucleation, growth and stabilisation of QDs. Additionally, sulphide anions (S^2-^) may have electrostatically interacted with -NH_3_^+^ groups of chitosan during the synthesis of ZnS QDs at lower pH, which could also affect the sizes of the nanocrystals formed.

In addition, photoluminescence properties were also affected by pH. The PL relative efficiency of the CHI-ZnS bioconjugates was higher under more acidic synthesis conditions (pH = 4.0). PL quenching may be attributed to several features. In this case, at relatively higher pH levels (pH = 5.0 and pH = 6.0), the smaller sizes of the nanoparticles were observed, and most of the amine groups were deprotonated (pH closer to pKa). As the nanoparticle size decreases, surface disorder and dangling bonds may dominate the luminescence properties, thus creating non-radiative pathways that dissipate quantum dot emission, which resulted in the decreased PL intensity [56,57]. Considering spherical quantum dots, as the nanoparticle size reduces (radius, *R*), the relative surface (*S*) to volume (*V*) ratio (*S*/*V* = 4*πR*^2^ / (4/3)*πR*^3^) = 3/*R*) is significantly increased leading to more surface defects. Additionally, amine groups can act as hole scavengers, which quench the photoluminescence [58].

## Conclusions

In the present work, ZnS QDs directly biofunctionalised by chitosan were synthesised using a single-step colloidal process in aqueous medium at room temperature. The results demonstrated that varying the pH from 4.0 to 6.0 of the chitosan solutions significantly affected the average size of ZnS nanocrystals produced ranging from 3.8 to 4.7 nm. The results indicated the stabilisation of ZnS conjugates by the interaction of the functional groups of chitosan, amines, acetamides and hydroxyls, with Zn^2+^ at the surface of QDs. Additionally, the pH of the solution induced important effects on the optical fluorescent behaviour of the ZnS-chitosan bioconjugates which was assigned to the 'trap states’ emissions involving the defect states of the QDs. Hence, new cadmium-free biocompatible colloids based on ZnS QDs capped by chitosan were successfully developed exhibiting luminescent activity that may be tuned by adjusting the pH with great potential for use in biomedical and eco-friendly applications.

## Competing interests

The authors declare that they have no competing interests.

## Authors’ contributions

HSM carried out the experimental design and analysis and drafted the manuscript. AAPM carried out the characterization and analysis and drafted the manuscript. FPR participated in the synthesis, characterization and analysis of quantum dots. All authors read and approved the final manuscript.

## Supplementary Material

Additional file 1: Figure S1 Infrared spectra of chitosan (pH = 4.0). Inset: vibrational region: 1,750 to 1,400 cm^-1^. Click here for file

Additional file 2: Figure S2 FTIR spectra of CHI (a) and CHI-ZnS (b) at pH = 5.0 ± 0.2. Vibrational regions: 1,750 to 1,475 cm^-1^ (left) and 1,250 to 950 cm^-1^ (right). Click here for file

Additional file 3: Figure S3 FTIR spectra of CHI (a) and CHI-ZnS (b) in the range of 3,700 to 3,050 cm^-1^ at pH 6.0 ± 0.2 (A), pH = 5.0 ± 0.2 (B) and pH = 4.0 ± 0.2 (C). Click here for file

Additional file 4: Figure S4 Potentiometric titration curve of 75 mg of chitosan dissolved in 0.1 mol.L^-1^ HCl solution (a) and its derivative (b). Click here for file
