# Supplementary material for: One-step colloidal synthesis of biocompatible water-soluble ZnS quantum dot/chitosan nanoconjugates
Source: Nanoscale Res Lett. 2013 Dec 5;8(1):512. doi: 10.1186/1556-276X-8-512 (PMC4234014; doi:10.1186/1556-276X-8-512)
Supplement: Additional file 1: Figure S1 — Infrared spectra of chitosan (pH = 4.0). Inset: vibrational region: 1,750 to 1,400 cm-1. [file 1556-276X-8-512-S1.doc]

**Inset**

**Figure S1.** Infrared spectra of chitosan (pH = 4.0). Inset: vibrational region: 1,750 to 1,400 cm^−1^.
