# Supplementary material for: One-step colloidal synthesis of biocompatible water-soluble ZnS quantum dot/chitosan nanoconjugates
Source: Nanoscale Res Lett. 2013 Dec 5;8(1):512. doi: 10.1186/1556-276X-8-512 (PMC4234014; doi:10.1186/1556-276X-8-512)
Supplement: Additional file 2: Figure S2 — FTIR spectra of CHI (a) and CHI-ZnS (b) at pH = 5.0 ± 0.2. Vibrational regions: 1,750 to 1,475 cm-1 (left) and 1,250 to 950 cm-1 (right). [file 1556-276X-8-512-S2.doc]

Wavenumber (cm^-1^)

**Figure S2.** FTIR spectra of CHI (a) and CHI-ZnS (b) at pH = 5.0 ± 0.2. Vibrational regions: 1,750 to 1,475 cm^−1^ (left) and 1,250 to 950 cm^−1^ (right).
