# Supplementary material for: One-step colloidal synthesis of biocompatible water-soluble ZnS quantum dot/chitosan nanoconjugates
Source: Nanoscale Res Lett. 2013 Dec 5;8(1):512. doi: 10.1186/1556-276X-8-512 (PMC4234014; doi:10.1186/1556-276X-8-512)
Supplement: Additional file 3: Figure S3 — FTIR spectra of CHI (a) and CHI-ZnS (b) in the range of 3,700 to 3,050 cm-1 at pH 6.0 ± 0.2 (A), pH = 5.0 ± 0.2 (B) and pH = 4.0 ± 0.2 (C). [file 1556-276X-8-512-S3.doc]

(A)

(B)

(C)

**Figure S3.** FTIR spectra of CHI (a) and CHI-ZnS (b) in the range of 3,700 to 3,050 cm^−1^ at pH 6.0 ± 0.2 (A), pH = 5.0 ± 0.2 (B) and pH = 4.0 ± 0.2 (C).
