# Supplementary material for: One-step colloidal synthesis of biocompatible water-soluble ZnS quantum dot/chitosan nanoconjugates
Source: Nanoscale Res Lett. 2013 Dec 5;8(1):512. doi: 10.1186/1556-276X-8-512 (PMC4234014; doi:10.1186/1556-276X-8-512)
Supplement: Additional file 4: Figure S4 — Potentiometric titration curve of 75 mg of chitosan dissolved in 0.1 mol.L-1 HCl solution (a) and its derivative (b). [file 1556-276X-8-512-S4.doc]

(a)

(b)

**Figure S4.** Potentiometric titration curve of 75 mg of chitosan dissolved in 0.1 mol.L^−1^ HCl solution (a) and its derivative (b).
